# Marine heatwaves in the Humboldt current system: from 5-day localized warming to year-long El Niños

**DOI:** 10.1038/s41598-021-00340-4

**Published:** 2021-10-27

**Authors:** Alice Pietri, François Colas, Rodrigo Mogollon, Jorge Tam, Dimitri Gutierrez

**Affiliations:** 1 Instituto del Mar del Peru (IMARPE), Callao, Peru; 2 LOCEAN-IPSL, IRD/Sorbonne Université/CNRS/MNHN, Paris, France; 3 Laboratorio de Ciencias del Mar, Universidad Peruana Cayetano Heredia, Lima, Peru

**Keywords:** Ocean sciences, Physical oceanography, Ocean sciences

## Abstract

During the last 4 decades punctual occurrences of extreme ocean temperatures, known as marine heatwaves (MHWs), have been regularly disrupting the coastal ecosystem of the Peru-Chile eastern boundary upwelling system. In fact, this coastal system and biodiversity hot-spot is regularly impacted by El Niño events, whose variability has been related to the longest and most intense MHWs in the world ocean. However the intensively studied El Niños tend to overshadow the MHWs of shorter duration that are significantly more common in the region. Using sea surface temperature data from 1982 to 2019 we investigate the characteristics and evolution of MHWs, distinguishing events by duration. Results show that long duration MHWs (> 100 days) preferentially affect the coastal domain north of 15° S and have decreased in both occurrence and intensity in the last four decades. On the other hand, shorter events, which represent more than 90% of all the observed MHWs, are more common south of 15° S and show an increase in their thermal impact as well as on the number of affected days, particularly those spanning 30–100 days. We also show that long duration MHWs variability in the coastal domain is well correlated with the remote equatorial variability while the onset of short events (< 10 days) generally goes along with a relaxation of the local coastal wind.

## Introduction

Marine heatwaves (MHWs) are characterized by prolonged extreme warming of the ocean and can have important effects on marine ecosystems. Atmospheric heatwaves have been intensively studied during the past decades since global warming has been shown to increase their frequency and intensity on land^1^. As a result, extreme warming events in the ocean, where most of the global excess heat is stored, have also started to draw attention in the recent years^2,3^. Although there is no universal definition for a MHW, Hobday et al.^4^’s method, based on atmospheric studies, has been widely used in the past few years for comparative studies, occasionally tuned and tweaked for regional specificities^5–7^. This allowed for direct comparison between events characteristics such as duration, intensity and spatial extension among others in different areas of the world’s ocean.

Driven by climate change, upper ocean temperatures have warmed significantly in most regions of the world over recent decades^8,9^. Along with this long-term warming signal, the frequency and intensity of extreme temperature events are also increasing^10,11^. In fact the amount of days affected yearly by MHWs have been shown to have increased by $54\%$ since 1925, a trend that tends to accelerate over the last decades^12^. This increase is mainly due to a globally warmer ocean but also to an increase in its temperature variability^13^.

Coastal regions are particularly sensitive to changes in ocean temperature because their environmental and socio-economic developments are often very dependent on the variability of marine resources. In this context, the dramatic ecosystem changes that have been reported in association with extreme marine heatwaves^14,15^ can have devastating consequences. In particular, Eastern Boundary Upwelling Systems (EBUS) are some of the most productive regions in the world ocean^16^ and the dynamics that sustains such productivity is closely linked to the relatively cold coastal surface temperature rendering them sensitive to extreme warming events. Among those regions, the Peru Chile Upwelling System (PCUS) is characterized by year-long coastal upwelling that makes it the most productive of all coastal upwelling systems^17^. Besides, its proximity to the equator makes it also highly sensitive to equatorial waves activity and to the regular occurrences of El Niño (EN) events that manifest as individual, long-lasting MHWs^18^. In fact, Oliver et al.^13^ showed that the eastern equatorial Pacific ocean is one of the hot spots of high MHW intensity and duration.

Even though, during the last few years, several studies have focused on the characteristics of MHWs, there is still a limited understanding of the physical processes that trigger them. Based on a review of the literature, Holbrook et al.^3^ conducted a study on MHWs drivers and concluded that basin-scale climate variability plays a significant role in the frequency of occurrence and intensity of these events. In particular, El Niño Southern Oscillation (ENSO) exhibits a large area of influence, enhancing (suppressing) MHW occurrence in the eastern Pacific Ocean in its positive (negative), El Niño (La Niña) phase. Although, Holbrook et al.^3^ showed that the PDO mostly influence MHWs in the North Pacific, it also modulates the frequency and intensity of ENSO cycle on decadal time scales. In the southern part of the PCUS, interannual modes of variability have been discussed such as the South Pacific Meridional Mode (SPMM^19^) or the “Chile Niño”^20^. They are related to positive feedbacks between SST and wind forcing and could lead to warm SST anomalies that would favor the development of MHWs.

Climate modes of variability significantly influence the occurrence of MHWs at interannual to decadal time scales, but the generation of MHWs is also strongly related to teleconnections at intraseasonnal time scales, and regional air sea coupling^21^. In fact, the PCUS is subject to important variability due to local and remote forcing: it is influenced by equatorial wave activity in the form of Intraseasonal Equatorial Kelvin Waves (IEKW^22^) that reach the South American coast and transmit part of their energy to southward propagating coastal-trapped waves (CTW)^23–25^. It is also sensitive to the variability of the alongshore wind, whether locally or through the generation of CTW at remote locations along the coast^25–27^.

The significant progress in the global understanding of MHWs highlights the need for more regional studies to better understand local processes and impacts. Varela et al.^28^ studied MHWs in EBUS and showed that since the 80s the trend for an increase in MHW occurrences is reduced in the coastal region, where cold upwelled waters are found, compared to the open ocean. They also pointed out that this difference is less important in the Peru region and that it is the only EBUS where there is a trend for a reduction of MHW occurrences. This specificity of the PCUS is most likely the result of ENSO variability which tends to hide variability of more localized events of shorter duration.

Considering the impact MHWs have on marine ecosystems, a dedicated study aiming to disentangle EN variability from other type of MHWs to investigate their respective properties and evolution is of utmost importance. To this end, satellite sea surface temperatures (SST) from 1982 to 2019 are used in the present work to characterized MHWs and their trend in the PCUS with a classification of events by range of duration. Furthermore we also explore the remote and local origin of MHWs and discuss their possible drivers.

## Results

### Marine heatwaves in the coastal domain from 1982 to 2019

**Figure 1:**
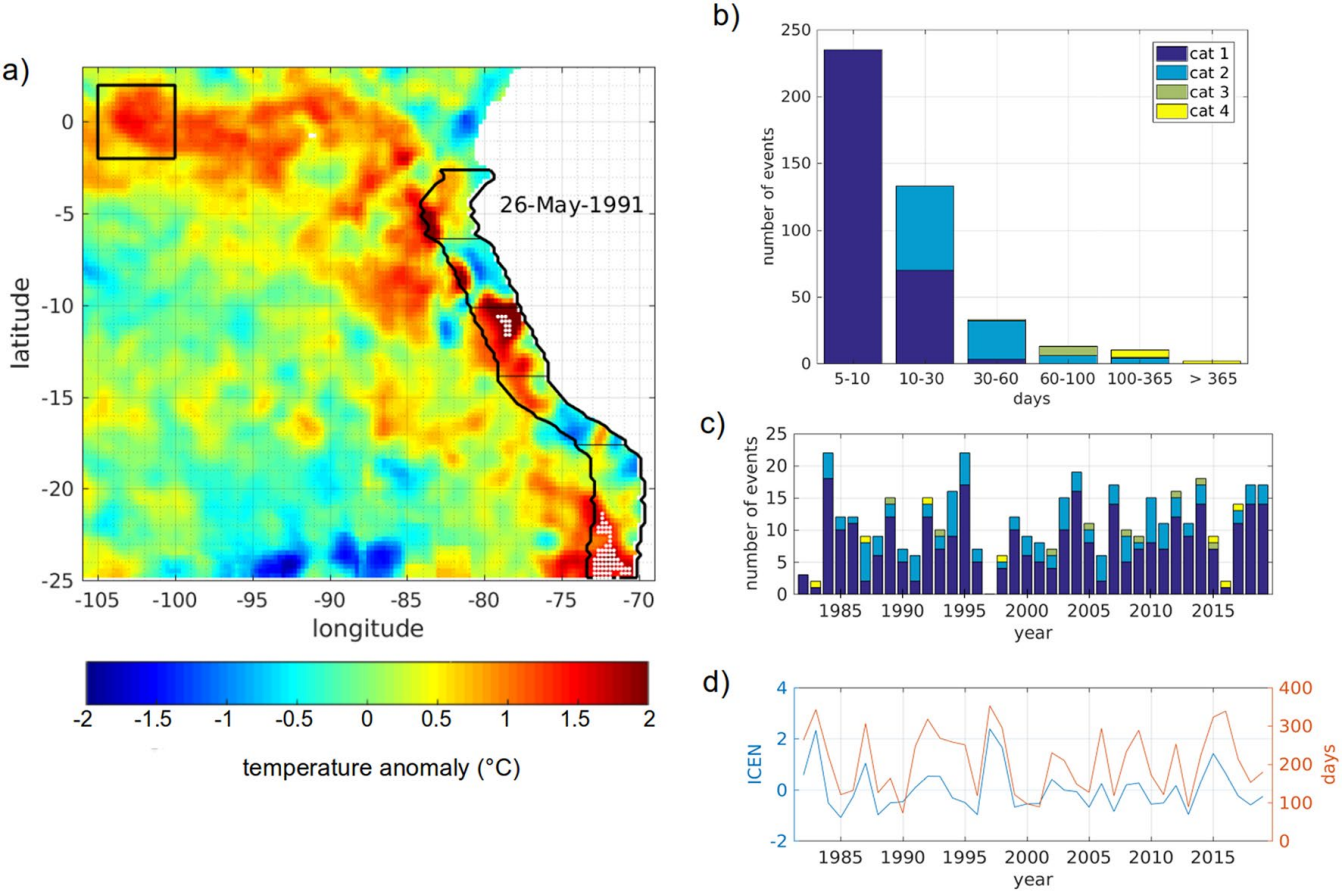
(**a**) SST anomaly in the South Eastern Pacific on 26 May 1991. The coastal domain considered in the study is circled in black. The black box represents the equatorial region used to detect remote occurrences of MHWs. White dots indicate data point where the criterium for MHW detection is met. Agglomerations of white dots at $\sim 11^{\circ}\,\hbox{S}$ and $\sim 23^{\circ}\,\hbox{S}$ correspond to two spatially coherent MHW events. Histograms on the right side show the distribution of MHWs in the coastal region. (**b**) Number of MHWs sorted by duration and category. (**c**) Number of events each year, colors indicate the category in which they are. When an event spans over 2 years it is allocated to the year where the maximum SST anomaly falls. (**d**) ICEN values averaged yearly (blue) and number of days affected yearly by MHWs in the coastal domain (orange). The figure was created using Matlab R2020b (https://matlab.mathworks.com/).

Over the 38-year SST time series, 426 MHWs were identified on the coastal band up to $2.5^{\circ }$ off the coast, they range from 5 to 610 days in duration and spatially cover from 1 data point ($\sim 625\,\hbox {km}^2$) to the whole coastal domain ($\sim 562{,}500\,\hbox {km}^2$). About $90\%$ of the total events, 368 over 426, last less than 30 days (Fig. 1b). On average the coastal domain is subjected to 11 events a year with a maximum of 22 events observed in 1984 and 1995 (Fig. 1c). The number of MHW days in a year identified in the coastal domain ranges from 73 days in 1990 to almost the whole year when long duration MHWs are observed (Fig. 1d). Those long duration events tend to be associated with EN. In fact there is a correlation of 0.84 between the number of MHW days in a year and the index ICEN (Fig. 1d).

**Figure 2:**
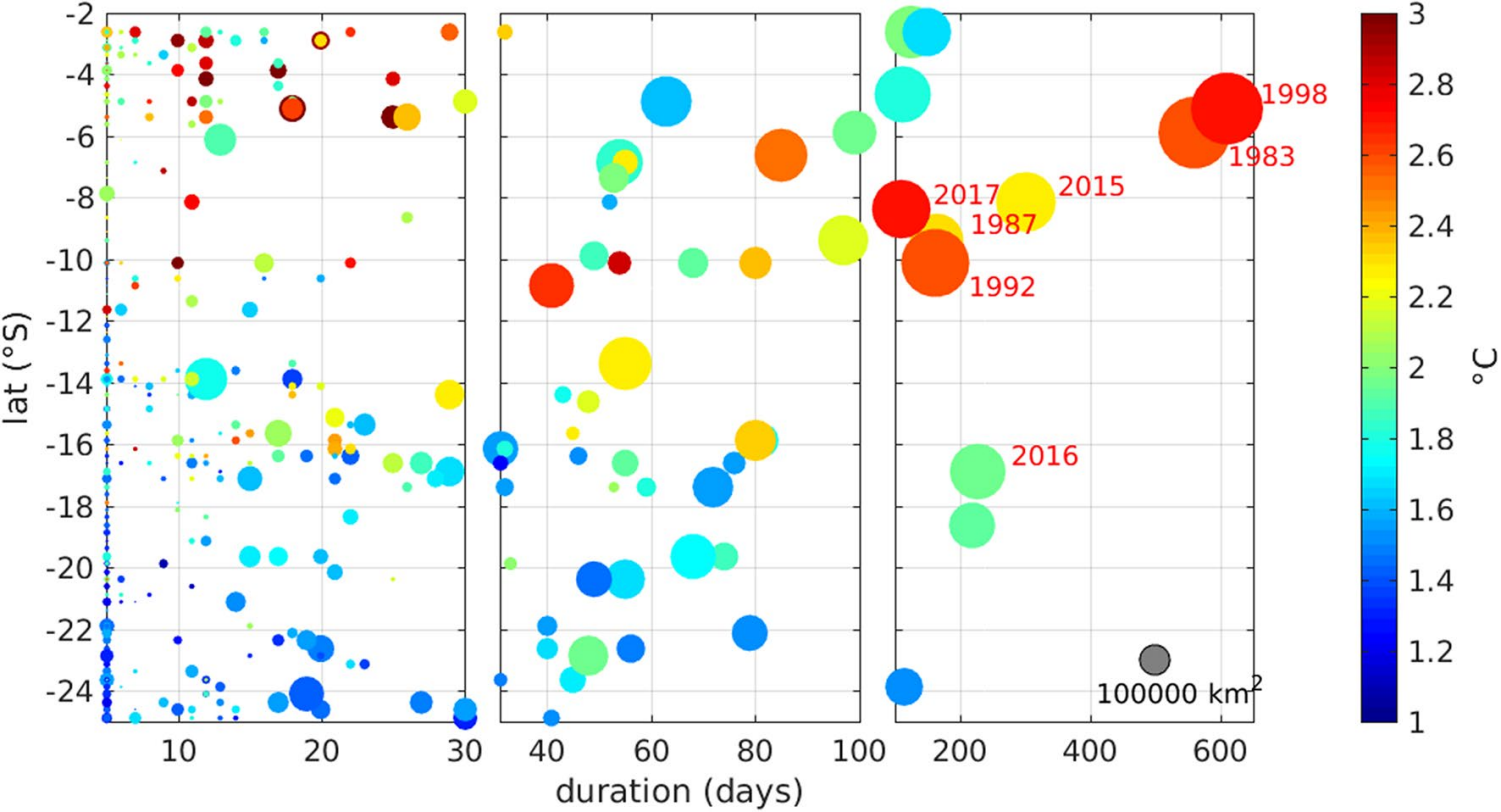
Scatter plot of MHWs duration organized by latitude. The dot size is function of the maximum surface occupied by the MHW on one day (the grey dot indicates a $10^6$ km^2^ MHW) while the color corresponds to the surface temperature anomaly averaged over the duration of the event. Years are indicated for all category 4 MHWs. The figure was created using Matlab R2020b (https://matlab.mathworks.com/).

MHWs shorter than 10 days all fall in category 1 (Fig. 1b) while category 2 MHWs range from 10 to 142 days and category 3 from 41 to 219 days. The fourth category almost only includes MHWs that correspond to El Niño events (Fig. 1c,d). In fact all category 4 events last more than 100 days and in particular two of them last more than a year. They correspond to the extremes EN of 82–83 (560 days MHW from 05 May 1982 to 16 November 1983) and 97–98 (610 days MHW from 01 January 1997 to 15 September 1998). Category 4 events are highlighted by their year on Fig. 2, most of them are driven by occurrences of EN and their epicentre, where the highest temperature anomaly is recorded, is located in the northern part of the domain. The only exception is the 2016 MHW which, even though it occupies the whole coastal band, is intensified between 15 and $20^{\circ}\,\hbox{S}$ and is not associated with a particularly high ICEN number ($ICEN\sim 0.6$ on average in 2016, Fig. 1d).

**Figure 3:**
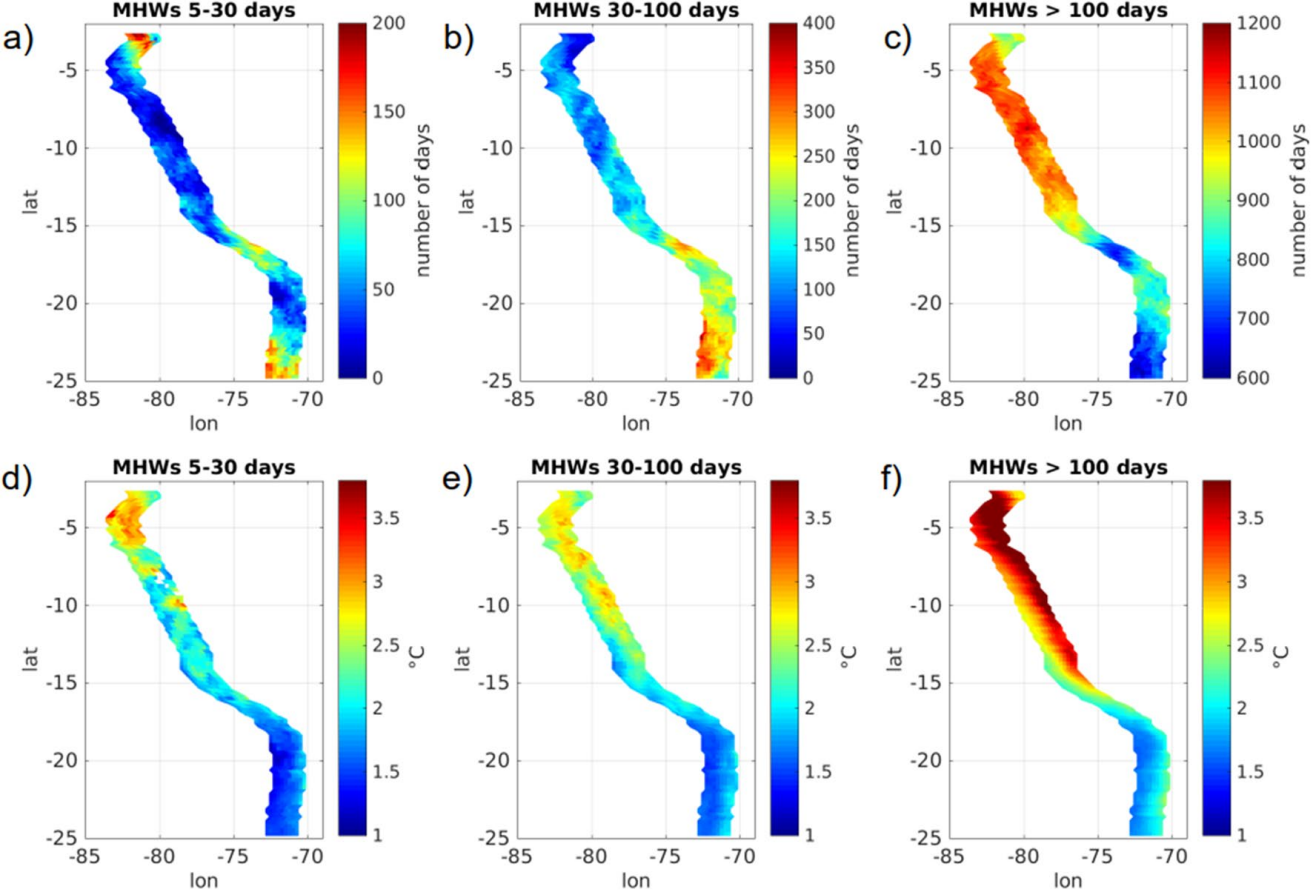
(**a**–**c**) Exposure to MHWs, expressed as the number of days affected on each data point and (**d**–**f**) averaged SST anomaly for MHWs of duration (**a**, **d**) 5–30 days, (**b**, **e**) 30–100 days and (**c**, **f**) longer than 100 days. The figure was created using Matlab R2020b (https://matlab.mathworks.com/).

The spatial distribution of MHWs characteristics is contrasted from north to south. Figure 2 illustrates the events duration, size and intensity by latitude. Shorter events (5- to 30-day long) tend to be small and relatively less intense, they are preferentially found south of $12^{\circ}\,\hbox{S}$. In fact only three MHWs of more than 80 days can be found in the southern half of the coastal domain. Intensity of MHWs clearly increases northward for all events duration but MHWs of less than 30 days are particularly intense within the nothernmost coastal region ($2.5^{\circ }{-}6^{\circ}\,\hbox{S}$). Maps of exposure (number of days affected by a MHW during the 38 years of the dataset, 3a–c) confirm that the southern part of the domain is more prone to be affected by short and small size events while longer and warmer ones develop preferentially in the northern part. In fact the exposure to 5- to 100-day MHWs is higher south of $14^{\circ}\,\hbox{S}$ with preferred locations around $16^{\circ }{-}17^{\circ}\,\hbox{S}$ and $22^{\circ }{-}25^{\circ}\,\hbox{S}$ (Fig. 3a,b). MHWs of more than 100 days are more common in the northern part and the exposure decreases significantly south of $14^{\circ}\,\hbox{S}$. Regardless of the duration, MHWs temperature anomaly is higher north of $14^{\circ}\,\hbox{S}$, with a $2\,^{\circ }\hbox {C}$ averaged anomaly than south of $14^{\circ}\,\hbox{S}$ with a $1.6\,^{\circ }\hbox {C}$ averaged anomaly (Fig. 3d–f and Table 1). Overall, in the northern part of the domain the exposure to the longest MHWs is about 7 times higher than the exposure to the shorter ones while in the southern part it is only twice as high (Fig. 3a–c). Those very large scale and long duration events tend to hide the variability and evolution of smaller events that are significantly more frequent. In fact, up to 20 events can happen yearly on the coastal domain, and are as such worthy of a special attention (Fig. 1c).

**Table 1:** Characteristics of MHWs in the coastal domain from 1982 to 2019.

Latitude range	Number of events	Average size ($10^4\,\hbox {km}^{2}$)	Average duration (days)	Average SST anomaly ($\,^{\circ }\hbox {C}$)
2.5–$6^{\circ}\,\hbox{S}$	99	3.59	26	2.22
6–$10^{\circ}\,\hbox{S}$	27	8.89	43	2.10
10–$14^{\circ}\,\hbox{S}$	55	3.41	16	2.01
14–$18^{\circ}\,\hbox{S}$	102	2.42	20	1.80
18–$25^{\circ}\,\hbox{S}$	143	2.12	15	1.54

**Figure 4:**
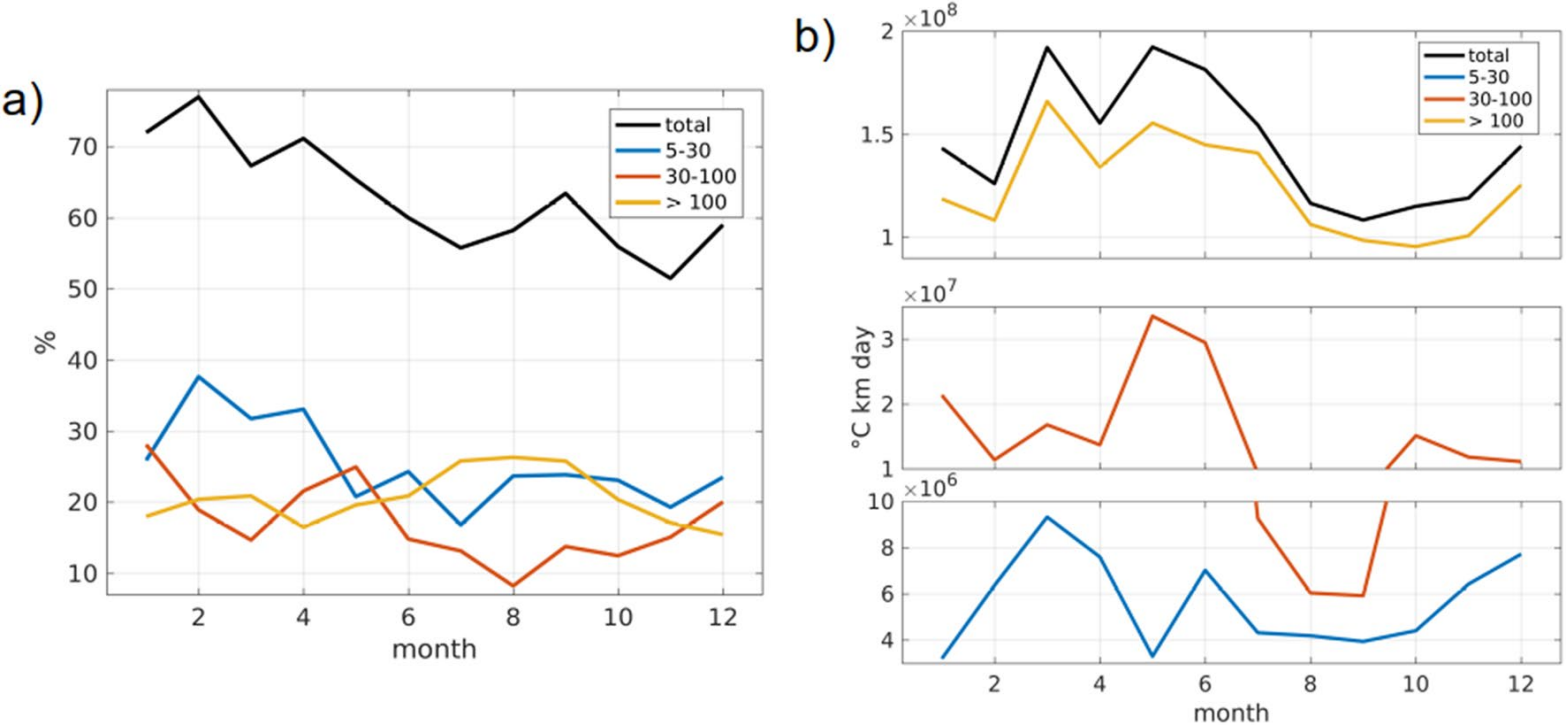
(**a**) Monthly percentage of exposure, i.e. proportion of days affected by a MHW anywhere in the coastal domain and, (**b**) resulting net heating, i.e. SST anomaly summed over space and time. Results are presented for all events (black), events of duration 5–30 days (blue), 30–100 days (red) and longer than 100 days (yellow). The figure was created using Matlab R2020b (https://matlab.mathworks.com/).

The monthly percentage of exposure to MHWs is presented Fig. 4a. In general MHWs tend to affect the coastal domain preferably from January to April, however this seasonality is not uniform among MHWs depending on their duration. Note that if shorter MHWs have a lower exposure locally (Fig. 3), when considering how often they occur somewhere over the whole domain they tend to affect more days than longer events. The maximum frequency in February is mainly driven by shorter events, in fact about half of the events that occurred in February lasts less than 30 days. From February to April the probability that at least one 5- to 30-day MHW would be observed on the domain is more than $30\%$. This is coherent with the peak in intraseasonal variability found in summer for the SST^26,29^. On the contrary, long events tend to occur preferably in austral winter, from July to September.

A value for the net heating associated with MHWs for every month of the year was then computed. This value is a good indicator for the impact that a MHW can have on the entire coastal strip since the highest values will be associated with a large spatial extension, a long duration and intense SST anomalies. Seasonality of net heating showed the most pronounced effect from March to August. This variability is largely related to long duration events that drive large heating in austral autumn and winter (March–July, Fig. 4b). Note that net heating seasonality differs from exposure seasonality which means that events with large persistence probably exhibit highest SST anomalies or affect larger areas in autumn than they do in winter. From May to June the heating due to 30- to 100-day MHWs is the highest, while 5- to 30-day MHWs have the highest impact in March–April.

### Evolution during the last 4 decades

**Figure 5:**
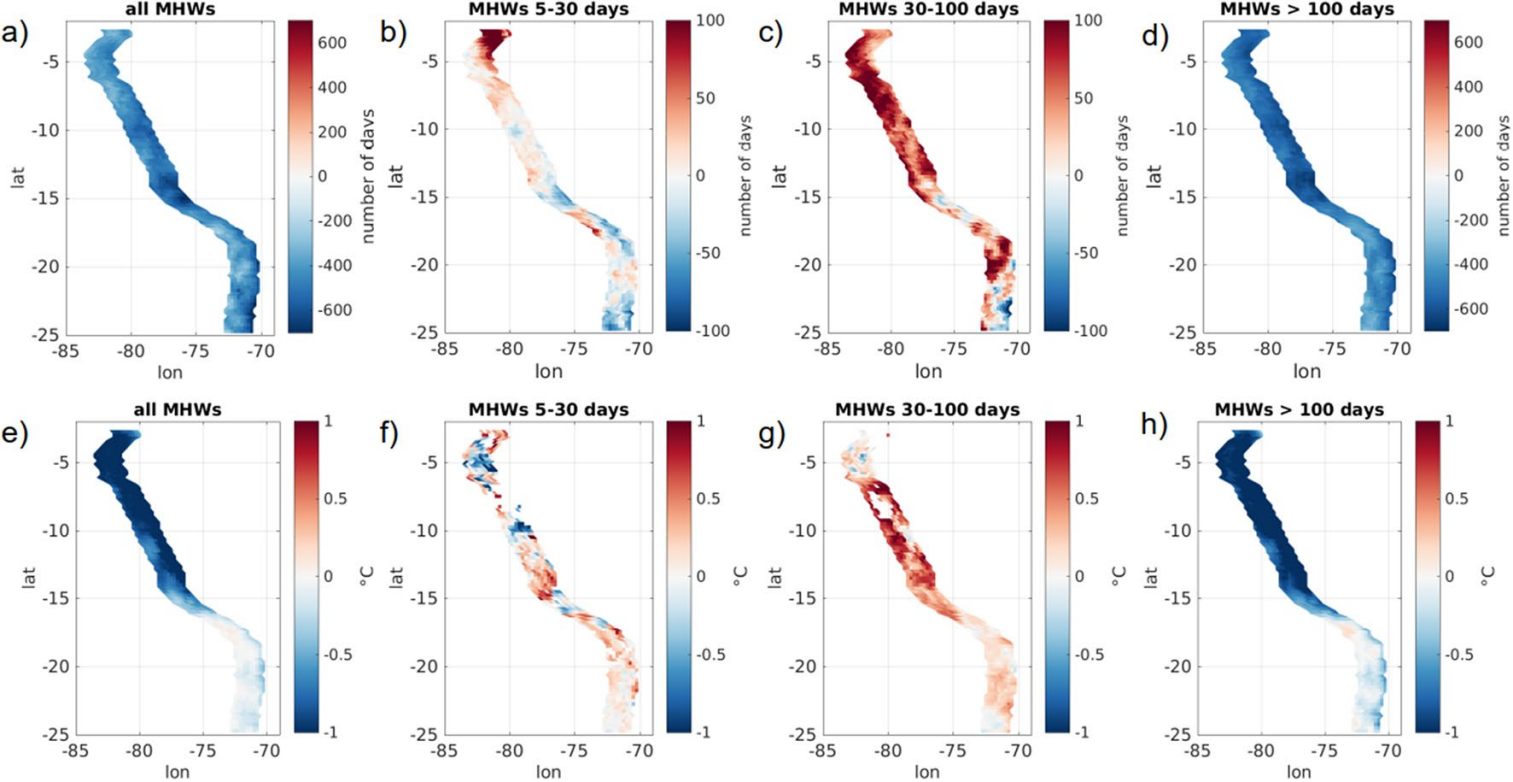
Differences in MHWs exposure and intensity between the period 1982–2000 and the period 2001–2019 (calculated as [2001:2019]–[1982:2000]). (**a**–**d**) Difference in the number of days affected on each data point and (**e**–**h**) difference in averaged intensity for (**a**, **e**) all events, events of duration (**b**, **f**) 5–30 days, (**c**, **g**) 30–100 days and (**d**, **h**) longer than 100 days. The figure was created using Matlab R2020b (https://matlab.mathworks.com/).

The evolution of MHWs is studied by comparing two 19-year time periods, 1982–2000 and 2001–2019, in a similar manner as was done by Oliver et al.^12^ i.e. looking at average exposure and intensity for MHWs in the 2001–2019 time range minus these same values in the 1982–2000 interval. In general MHW exposure shows a decrease in the recent period compared to the earlier one along with a reduction of the intensity (Fig. 5a,e), which is consistent with previous findings^12^. The average-465 days difference between the two periods can be explained by the presence of two majors EN events in 82–83 and 97–98 who drove MHWs of respectively 560 and 610 days. This also explains why the temperature anomaly associated with MHWs tends to be cooler in the recent period north of 16$^{\circ }$ S while in the southern part of the domain the trend is less pronounced. This evolution can also be related to variations of the PDO. In fact, the PDO is on average positive (warm) in the reference phase of the trend calculation ($\sim 0.1$ between 1982 and 2000), while it is negative (cold) in the more recent phase ($\sim -0.5$ between 2001 and 2019) which could contribute to the reduction of MHW occurrence and intensity observed between those two periods.

Looking at trends for exposure depending on the duration of MHWs allows to highlight different variabilities. In fact the tendency to a reduction in exposure and intensity is mainly driven by long duration events (Fig. 5d,h), which was expected considering the strongest EN are found in the first interval of time (Fig. 1d). Evolution of events with duration 30–100 days follows an opposite trend (Fig. 5c), the number of days affected by events in this duration range increased over the last decades with, on average, 46 more days during the period 2001–2019 compared to the period 1982–2000. There is an asymmetrical response of short lived MHWs ($< 30\,\hbox {days}$) north and south of $15^{\circ}\,\hbox{S}$, with 23 more days observed in the recent period in the northern region while the southern part of the domain has seen 14 days less (Fig. 5b). Intensity evolution also shows a contrasted picture depending on event duration. While long duration events ($> 100\,\hbox {days}$) have been colder by $0.7\,^{\circ }\hbox {C}$ on average in the recent decades it is not the case for shorter events. Events 30–100 days exhibit on average an intensity $0.3\,^{\circ }\hbox {C}$ higher (Fig. 5g) and events shorter than 30 days don’t show any consistent signal in intensity change along the coast but only localized variability (Fig. 5f).

**Figure 6:**
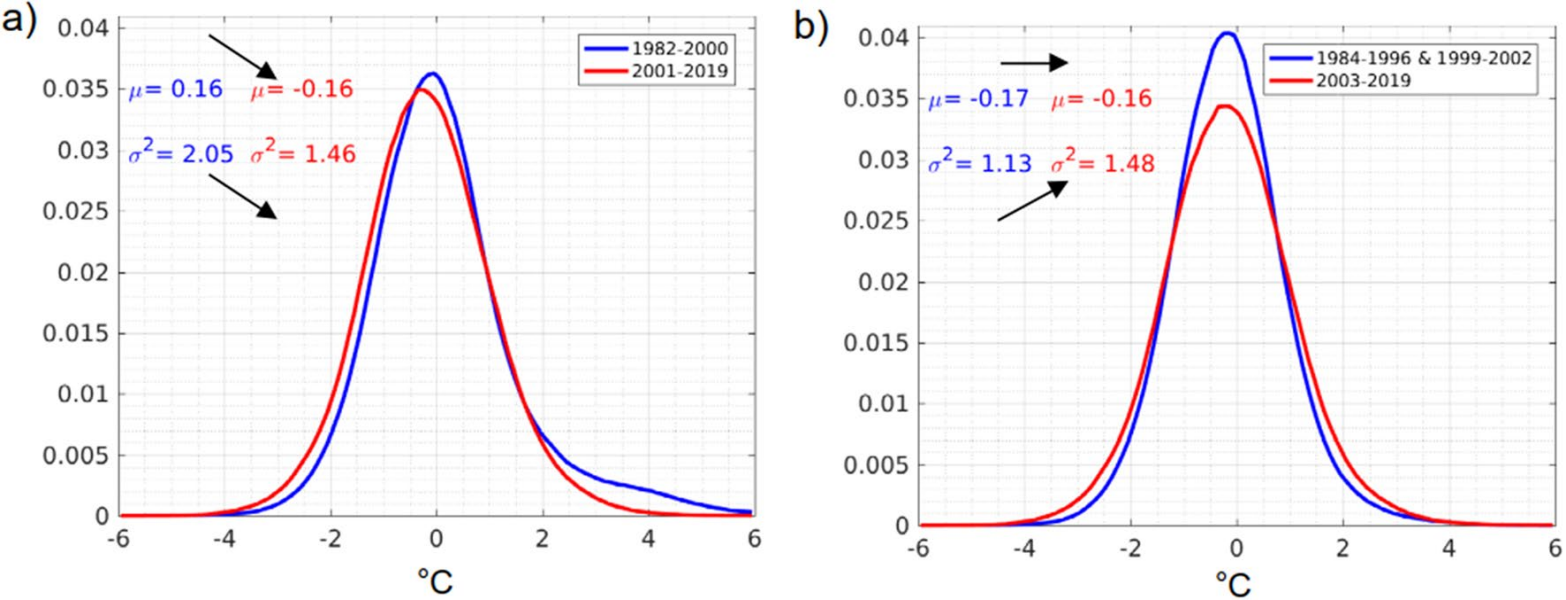
Probability density functions of daily SST anomalies for two periods of equal duration. The blue line represents the first half of the distribution: (**a**) 1982–2000 or (**b**) 1983–1996 and 1999–2002. The red line represent the second half: (**a**) 2001–2019 or (**b**) 2003–2019. The mean ($\mu$, in degrees Celsius) and standard deviation ($\sigma$, in degrees Celsius) are also indicated for the two time periods. The figure was created using Matlab R2020b (https://matlab.mathworks.com/).

More frequent and/or intense MHWs can be the result of two conditions: the increase of the mean SST and/or the increase of the SST variance^30^. In the Eastern South Pacific, linear regression of the SST time series since the 80s tend to show a reduction of the mean SST^31^. In fact, comparing the SST distribution for the periods 1982–2000 and 2001–2019, the recent years are $\sim 0.3 \,^{\circ }\hbox {C}$ cooler with a variance $\sim 0.6\,^{\circ }\hbox {C}$ lower (Fig. 6a). However, considering that the El Niños of 82/83 and 97/98 have a strong impact on the evolution of the SST in the last 4 decades we removed those years and compared the distributions again: when discarding those four years and comparing the period 1984–1996 plus 1999–2002 to the period 2003–2019 (two 17 years periods) the difference in SST is almost null but the difference in variance shows an increase of $0.35 \,^{\circ }\hbox {C}$ (Fig. 6b). Oliver et al.^30^ showed that the global trend to an increase in MHWs frequency and intensity was driven by the mean SST and not the variance almost everywhere except in the highly variable western boundary currents. Marin et al.^32^ however, focusing only on the very coastal region, showed that in the PCUS the general trend for a decrease in duration and intensity of MHWs was mostly driven by internal variability of the SST. We show here that, by removing the four years of the 38-year time series that correspond to the strongest EN conditions, the tendency for increased occurrence of MHWs shorter than 100 days in the PCUS might be the result of a higher SST variability. Note that Fig. 6b also shows that there is a highest probability of occurrence for marine cold-spells.

Thus, even though at first glance the evolution of MHWs in the Eastern South Pacific seems to follow an inverse trend compared to the global ocean, it is mainly driven by few very large scale events (see Oliver et al.^12^ supplementary information on the spatial influence of ENSO on the mean and variability of MHW properties). Shorter events variability is on par with the global trend for increased exposure. This is an important result with implications on the coastal environment since about $70\%$ of MHWs shorter than 100 days are located less than 100 km from the shore.

### Local and remote origin of marine heatwaves

Anomalous warm ocean temperatures can emerge for various reasons, however in the PCUS it is mainly driven by remote equatorial variability that can drive advection of warm temperatures, equatorial waves activity that propagate and modify the coastal stratification or local air-sea interactions through the relaxation of the coastal wind.

**Figure 7:**
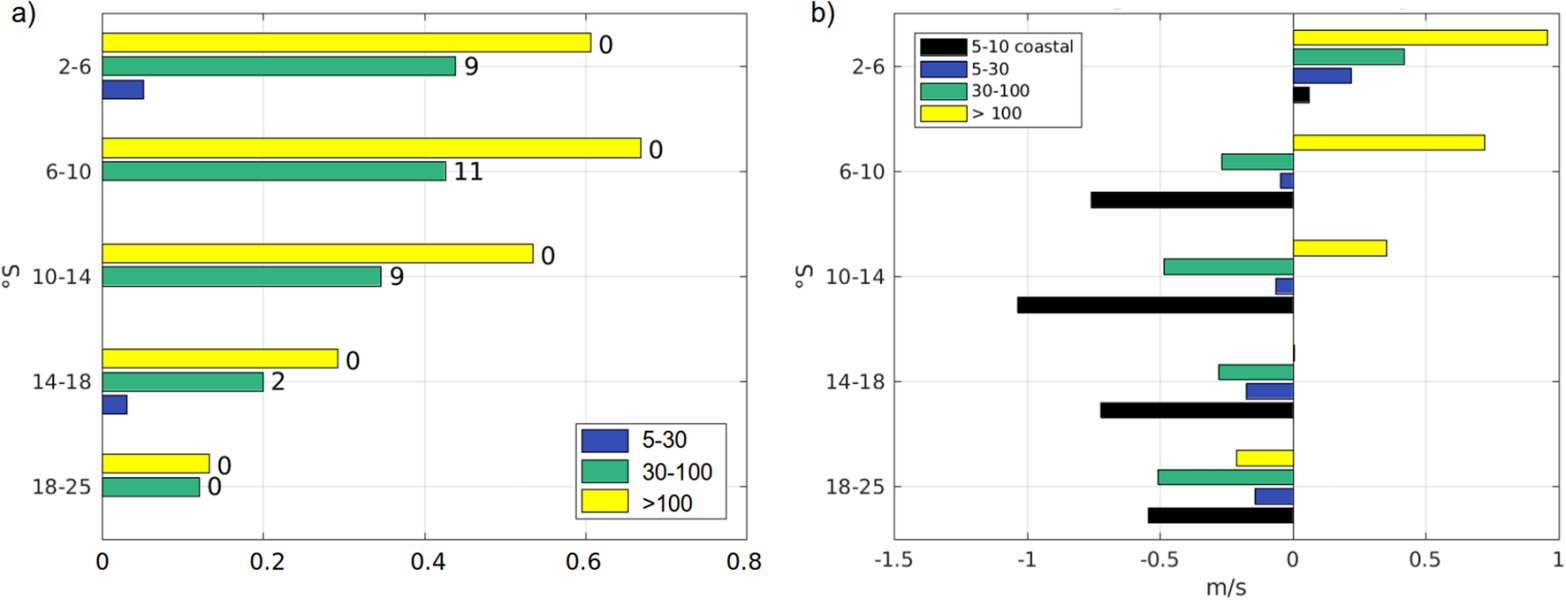
(**a**) Correlation between equatorial MHWs and coastal MHWs for 5 coastal latitude bands. The correlation is presented for events of duration 5–30 days (blue), 30–100 days (green) and longer than 100 days (yellow). The number indicates the lag for the highest correlation, in days. (**b**) Averaged wind anomaly on the first day of a MHW event. Results are presented for events of duration 5–30 days (blue), 30–100 days (green), longer than 100 days (yellow) and for events 5–10 days located less than 100 km from the shore (black). The figure was created using Matlab R2020b (https://matlab.mathworks.com/).

To gain insight on the influence equatorial MHWs can have on the emergence of MHWs in the coastal domain, the correlation between equatorial and coastal events of different duration is presented Fig.7a. Equatorial variability often propagates towards the east and along the South American coast in the form of equatorial Kelvin waves and CTW that can modify the SST by elevating or depressing the thermocline and affecting the entrainment of cold water into the mixed layer. In particular, downwelling waves deepen the thermocline leading to a warming of the SST. During EN events, advection of warm equatorial water by anomalous westerly winds has also been reported, although this process tends to be more pronounced in the western and central Pacific^33,34^.

The MHWs longer than 100 days show significant correlation ($> 0.5$), north of $14^{\circ}\,\hbox{S}$ for lags ranging from 0 to 30 days with the highest correlation found for zero lag. At longer lags, the correlation decreases. The good correlation at a large range of lags could be related to various processes propagating at different speed and acting together during EN events, resulting in a complicated phasing. In fact, in-phase variability of the SST along the equator was observed by Kessler et al.^35^ during the 1991/92 El Niño when warming and cooling events were shown to occur simultaneously over a wide longitude range. McPhaden et al.^33^ suggested that this could be explained by the different time it takes for SST to respond to zonal advection in the central Pacific and to vertical advection and entrainment in the eastern Pacific and by the difference in mixed layer depth in both regions. Since long MHWs are mostly related to strong EN events the same logic could apply and a mix of advection and entrainment acting on mixed layers of different depth could drive anomalously warm temperatures at the same time in the coastal and equatorial boxes.

MHWs with duration ranging from 30 to 100 days in the coastal domain also show significant correlation (0.35–0.45) with equatorial MHWs of the same duration north of $14^{\circ}\,\hbox{S}$ with a lag between the equator and the coast of about 10 days. Considering the distance between the equatorial and coastal boxes a 9–11 days lag would yield a propagation speed of the signal between 2.2 and 2.8 m $\hbox {s}^{-1}$. Since intraseasonal Kelvin waves, for time scales of $\sim 50{-}70\,\hbox {days}$, have been shown to propagate at $\sim 2.3{-}2.6 \,\hbox {m}\,\hbox {s}^{-1}$ they are a good candidate to explain the corresponding variability at the equator and at the coast^22^. In fact, comparing model outputs with different forcings from 2000 to 2008, Illig et al.^29^ showed that, in the coastal domain of central Peru, IEKW variability could account for $23\%$ of the SST variability in the $40-90\,\hbox {days}$ frequency band. This is coherent with the correlations between 0.12 and 0.44 found for events 30–100 days long along the coast. MHWs shorter than 30 days show very little to no significant correlation to the corresponding events in the equatorial region. South of $14^{\circ}\,\hbox{S}$, significant correlations are lower than 0.3 for all types of events.

Local wind conditions on the first day of a MHW were also investigated by latitude bands and for events of each duration range (Fig. 7b and Table 2). In the coastal strip, positive wind anomalies tend to be associated with cooling of the coastal waters through intensified coastal upwelling. Negative wind anomalies are generally associated with a slowing down of the equatorward wind and a warming of the near-surface waters. Figure 7b shows that, except for MHWs longer than 100 days, all of the other categories are on average accompanied by negative wind anomalies south of $6^{\circ}\,\hbox{S}$.

MHWs longer 100 days tend to exhibit positive wind anomalies north of $14^{\circ}\,\hbox{S}$ which is characteristic of the increase of the equatorward coastal wind observed during EN^36,37^. Chamorro et al.^38^ explained this coastal wind intensification by the important SST warming that occurs off northern Peru under EN conditions which tends to enhance the alongshore pressure gradient. This is coherent with the strong north-south gradient observed in SST anomaly distribution during long MHWs (Fig. 3f). Note that Fig.7b only shows the average wind on the first day of an event, however it is probable that the SST had started increasing before the onset until reaching the threshold, and thus the north-south temperature gradient may already exists at this time.

30- to 100-day MHWs present, on average, negative wind anomalies on the day of their onset, in the coastal subdomains south of $6^{\circ}\,\hbox{S}$. However the wind anomalies standard deviation is always larger than the (negative) value of the mean suggesting that local forcings are competing with other processes in this duration range. Note, however that all the distributions exhibit negative skewness which indicates the larger negative values are more common than the positive ones (Table 2).

In previous studies, the 5–30 days variability of the SST signal has been related with the synoptic atmospheric variability^26^ which would imply that at these time scales, SST warming is associated to weaker winds and a slowing down of the Ekman dynamics. In the coastal region, the wind stress energy has been shown to be mostly confined in the 2–40 days frequency band^29^. However, on average, south of $6^{\circ}\,\hbox{S}$ the wind anomaly observed on the onset of 5- to 30-day long MHWs is not strongly negative ($-0.34 \pm 1.38\hbox {m}\,\hbox {s}^{-1}$). This could be explained by the concomitant actions of wave activity and/or turbulent mesoscale variability that are not necessarily associated with a negative wind anomaly.

Among the MHWs shorter than 30 days, two additional categories of event where thus separated, the short MHWs (5–10 days) and the short and coastal MHWs (5–10 days and less than 100 km from the shoreline). Short duration events, that are more likely driven by local wind variability, present an averaged wind anomaly on day 1, south of $6^{\circ}\,\hbox{S}$, that is significantly more negative ($-0.75 \pm 1.54\hbox {m}\,\hbox {s}^{-1}$) than other categories. By removing offshore events we try and focus on MHWs that will have a significant impact on the coastal environment and remove anomalously warm situations associated with offshore propagating mesoscale eddies. Those short and coastal events are associated with the most negative wind anomalies from 6 to $14^{\circ}\,\hbox{S}$ (Table 2 and Fig. 7b).

**Table 2:** Statistics derived from the wind anomaly observed on the first day of a MHW. Statistics are shown in 5 coastal sub-domains and for different MHW duration. The category “5–10 days coastal” regroups MHWs that are located less than 100 km from the coast, the other categories are within $2.5^{\circ }$ of the shoreline. The last column indicates the number of grid point over which the average is calculated.

Latitude range	Duration range	Average wind anomaly ($\hbox {m}\,\hbox {s}^{-1}$)	Standard deviation ($\hbox {m}\,\hbox {s}^{-1}$)	Skewness ($\hbox {m}\,\hbox {s}^{-1}$)	Number of grid points
2.5–$6^{\circ}\,\hbox{S}$	5–10 days coastal	− 0.06	1.06	− 0.06	1593
	5–10 days	0.05	1.13	0.23	2173
	5–30 days	0.10	1.17	0.05	4407
	30–100 days	0.45	1.54	− 1.01	431
	> 100 days	1.41	0.55	− 2.07	308
6–$10^{\circ}\,\hbox{S}$	5–10 days coastal	− 0.74	0.62	− 1.43	312
	5–10 days	− 0.28	0.91	0.42	638
	5–30 days	− 0.23	1.16	− 0.51	1633
	30–100 days	− 0.12	1.20	− 0.06	670
	> 100 days	0.92	0.87	− 1.83	499
10–$14^{\circ}\,\hbox{S}$	5–10 days coastal	− 1.45	1.44	− 0.67	900
	5–10 days	− 0.94	1.59	− 0.38	1480
	5–30 days	− 0.30	1.64	− 0.35	2569
	30–100 days	–0.44	0.96	− 0.52	918
	> 100 days	0.58	1.23	− 1.34	502
14–$18^{\circ}\,\hbox{S}$	5–10 days coastal	− 0.46	1.53	− 1.00	1092
	5–10 days	− 0.64	1.47	− 0.57	1924
	5–30 days	− 0.23	1.32	− 0.45	4962
	30–100 days	− 0.31	1.02	− 0.61	1978
	> 100 days	− 0.11	0.86	− 0.34	675
18–$25^{\circ}\,\hbox{S}$	5–10 days coastal	− 0.62	1.41	− 0.19	1914
	5–10 days	− 0.80	1.62	0.12	3933
	5–30 days	− 0.44	1.37	− 0.26	8436
	30–100 days	− 0.59	1.40	− 0.04	3512
	> 100 days	− 0.09	0.63	− 0.93	1271

## Discussion

MHWs characteristics in the PCUS were studied using satellite sea surface temperature data between 1982 and 2019. It was shown that the larger and stronger events, that are generally associated with the warm phase of ENSO, dominate the signal. They preferentially affect the northern part of the domain (north of $15^{\circ}\,\hbox{S}$) where their intensity is significantly higher than in the southern part. Exposure to long duration events ($>100$ days) and the SST anomaly they produce have decreased in the recent period (2001–2019) compared to the period of reference (1982–2000). These results are coherent with several global studies of MHWs^12,13,18^ but they overshadow the variability of shorter events which actually constitute over 90% of MHWs.

MHWs shorter than 100 days, contrary to the longer ones, affect preferentially the southern part of the domain whereas their intensity is, similarly, higher in the north. 30- to 100-day MHWs had a higher thermal impact and affected more days in the recent decades over the whole coastal strip. MHWs shorter than 30 days exhibited a more localized variability, showing decreases or increases in exposure and intensity depending on the latitude. Our results highlight the importance to make the distinction between EN and shorter MHWs and illustrate the importance of regional studies when it comes to MHWs characteristics and evolution.

Previous studies showed that the dominant variability for SST in the coastal domain is 20–90 days with a peak at around 50 days^26,29^ which is coherent with the preferred duration of MHWs observed in the PCUS (Table 1 and Fig. 1b). In fact, those studies showed that the highest SST variability has been observed in austral summer^26,29^ when the largest number of 5- to 30-day MHWs were detected (Fig. 4a). Then it is not surprising that the increase in SST variability observed when discarding the years 82, 83, 97 and 98 was associated to an increase in the exposure to MHWs shorter than 100 days.

Two proxies were used to assess MHWs origins and possible drivers, (i) the correlation of MHW exposure at the equator and at the coast and (ii) the local wind anomaly at the start of each event. Using these two variables allowed us to draw certain conclusions on coastal MHWs and the conditions favorable for their development. In particular, equatorial variability is well correlated ($\sim 0.6$) with events of more than 100 days north of $14^{\circ}\,\hbox{S}$ which we related to Kelvin wave activity and more particularly to the generation of EN events. In the north, those events are also associated with positive wind anomalies probably due to the intensification of equatorward coastal winds that can be observed during EN^37,38^.

Kelvin wave activity and the subsequent CTW take place at a wide range of frequencies, from intraseasonal to interannual timescales and are probably the cause for the significant correlation with event 30–100 days. CTW have in fact been shown to affect the SST all along the coast of Peru and Chile as south as $30^{\circ}\,\hbox{S}$^25,39^, however there is a loss in correlation with the equatorial MHWs south of $14^{\circ}\,\hbox{S}$. This could be explained in part because some of the 30–100 days events can be driven by local atmospheric forcings, unrelated to equatorial activity. In fact, local wind anomaly observed on the first day of events shorter than 100 days is, on average, negative south of $6^{\circ}\,\hbox{S}$ with a significant variability around the mean (Table 2) that suggests this range regroups different types of MHWs that are not exclusively wind-driven.

Short-lived MHWs ($<30$ days) barely shows any correlation with equatorial variability but are generally associated with negative wind anomalies at the moment of their onset. However, in this duration range, there is also a large variability of the observed wind anomalies (Table 2). Mesoscale eddies with a positive signature in temperature could be classified as MHWs and would fall in the 5–100 days range. They would not be associated with specific wind anomalies and as such increase the variance of the wind anomaly distribution. Note that they could also smear the coastal wave signal and generate anomalously warm situations through eddy dynamics that would be unrelated to the equatorial forcing and decrease the correlation with equatorial MHWs in the 30–100 days range. Enhanced mesoscale activity has in fact been reported in the PCUS south of 15$^{\circ }$ S^40^. By only looking at MHWs close to the shoreline we probably eliminated part of the eddy-related signal and were able to increase the observed negative wind anomaly suggesting that those short-lived coastal events are mainly due to local wind relaxation. A dedicated study examining the causal relationship between the trend of the local wind and the trend of short-lived coastal MHWs is beyond the scope of the present study but we hope it will be addressed in future work.

The preferential relationships between long MHWs and equatorial variability, and short MHWs and local air-sea interaction do not hold for one specific very intense event in particular. In 2017 the northern Peruvian coast has been affected by an exceptional MHW that lasted 3 months, exhibited SST anomalies of more than $7\,^{\circ }\hbox {C}$ (Fig. 2). It was called a “coastal El Niño”^41^. In this case, while it was long lasting and particularly strong, downwelling equatorial Kelvin waves were not the cause of its formation while a local decrease of the winds in the eastern Pacific was observed^41–43^. This MHW could not be anticipated with the classical EN detection methods because it was not associated with equatorial SST anomalies, yet it brought strong precipitations, catastrophic flooding and landslides, pointing out the need for more tailored regional detection and forecasting methods for MHWs. In fact, disentangling local and remote variability of the coastal SST remains a challenge to this day and is the subject of many studies, all the more important in the context of global warming and the increase of extreme events occurrences in the ocean. Our analysis highlights the characteristic variability of events in the PCUS and the environmental conditions that accompany them according to their duration. Nevertheless the coastal system is a very dynamic environment where processes of different spatial and temporal scales overlap.

## Methods

### Detecting marine heatwaves

Daily satellite SST provided by the National Oceanic and Atmospheric Administration (NOAA) Optimum Interpolation SST (OISST) gridded at a $0.25^{\circ }$ resolution^44^ from 1982 to 2019 were used to study a coastal region along the western coast of South America. MHWs were detected and characterized in a $2.5^{\circ }$ wide coastal band that spans in latitude from $2.5^{\circ}\,\hbox{S}$ to $25.5^{\circ}\,\hbox{S}$. Additionally an equatorial box located at 100-$105^{\circ}\,\hbox{W}$ longitude and $2^{\circ}\,\hbox{S}{-}2^{\circ}\,\hbox{N}$ latitude was also used to compare MHWs occurrences there and in the coastal domain (Fig. 1a).

Using the SST daily time series on each point of the domain, the method for MHW identification described by Hobday et al.^4^ is followed: a MHW is detected when the SST rises above a threshold, determined by the $90{{\mathrm{th}}}$ percentile of the data distribution, for a duration of more than 5 days. Consecutive events separated by less than two days below the threshold are considered a continuous event. The climatology and threshold are calculated on every grid point of the coastal domain and for each day of the year using an 11-day window (averaged for the climatology, $90^{{\mathrm{th}}}$ percentile for the threshold). Hobday et al.^4^ recommend that smoothing of daily climatological time series should be performed. As such, a 30-day running mean is applied to the annual time series of the mean and threshold temperature values (see Hobday et al.^4^ for the complete description of the method).

SST time series in sub-domains are constructed by averaging the SST in five coastal boxes and an equatorial one. The five coastal sub-domains span the following latitude ranges: $2.5^{\circ }{-}6^{\circ}\,\hbox{S}$, $6^{\circ }{-}10^{\circ}\,\hbox{S}$, $10^{\circ }{-}14^{\circ}\,\hbox{S}$, $14^{\circ }{-}18^{\circ}\,\hbox{S}$ and $18^{\circ }{-}25^{\circ}\,\hbox{S}$. The equatorial box from 100 to $105^{\circ}\,\hbox{W}$ and from $2^{\circ}\,\hbox{S}$ to $2^{\circ}\,\hbox{N}$ (Fig. 1a). The same MHW detection algorithm is then used to identify events in the sub-domains and run correlation diagnostics.

### Identifying spatially coherent events

To spatially define a MHW in the coastal region the first criterion is that at least one grid point on the domain meets the conditions for MHW for more than 5 consecutive days. Similarly to the detection method applied to singular grid points, when consecutive spatial events are separated by less than two days they are considered a continuous event. This means that a spatially coherent event, of a duration of X days, can be made up of several grid points where events of a duration of Y days ($\hbox {Y} < \hbox {X}$) are detected. These data points that are affected concomitantly, at least partly, are therefore organized to form a longer event. However using only this criterion, concomitant MHWs spatially constricted on different parts of the domain, were counted as one single event, even though they could be events no larger than a few thousand of squared kilometers separated by several hundred of kilometers (see Fig. 1a for an example). This led to erroneous statistics on the duration and size of the MHWs in the local domain.

The spatial coverage of MHWs in regional studies is usually defined by the percentage of occupation of the domain (e.g. Darmaraki et al., 2019^6^ for the Mediterranean Sea) but in an elongated coastal fringe this definition is not ideal to characterize independent events. To circumvent this problem, each spatially detected MHWs is inspected for latitudinal gaps. That is to say, if there is a latitudinal band of at least 200 km width which is not under any MHW condition for the whole duration of the event, the domain is separated in two on both sides of this band. The detection algorithm is then applied separately on the two subdomains to identify the concomitant but not related MHWs. Figure 1a for example shows two separated regions under MHWs conditions, at $\sim 11^{\circ }$ S and $\sim 23^{\circ }$ S, that correspond to two distinct MHW events. This is a simple method that is well suited for a coastal fringe. Note that we do not expect there will be more than one independent event on the same latitude band considering the longitudinal extension of the domain does not exceed 300 km. When events are classified by duration, the classification is made using the spatially coherent events and not individual grid points.

To study MHWs seasonality we defined two metrics. The first one is the percentage of exposure, i.e. the percentage of days within a month that are affected by a MHWs anywhere in the coastal domain. The second one allows to quantify the net heating at monthly timescales and is obtained by summing the temperature anomalies over space and time for every MHWs within a month. When a MHW spans multiple months, a separate value is calculated for each affected month.

### Defining a category

A category is finally derived for each event in order to assess its environmental impact. Using the time series at each point, the SST anomaly is integrated over the whole MHW duration, resulting in a cumulative intensity which is then organized in 4 classes: category I events have a cumulative intensity lower than $25\,^{\circ }\hbox {C}\,\hbox {day}$, category II from 25 to $100\,^{\circ }\hbox {C}\,\hbox {day}$, category III from 100 to $200\,^{\circ }\hbox {C}\,\hbox {day}$ and category IV more than $200\,^{\circ }\hbox {C}\,\hbox {day}$. A spatially coherent event is categorized by the highest cumulative intensity observed over the affected area. This way of categorizing MHWs differs from the method of Hobday et al.^2^ who use the difference between the climatology and the threshold. While Hobday et al.^2^ use the local historical data at each point which has the advantage of using a threshold replicable everywhere in the ocean we decided on a more local classification. In fact, the cumulative intensity allows to take into account the duration of an event and to better assess the impact on the environment by giving as much weight to a short MHW with a strong peak as to a longer but less intense one.

### Wind

Daily surface winds (10 m) at $0.25^{\circ }$ horizontal resolution were extracted from ERA-Interim atmospheric reanalysis global product^45^ from 1982 to 2015. The wind is interpolated on the same grid as the SST data to provide statistics for MHWs. In order to derive wind anomalies, a climatology is calculated by averaging the zonal and meridional wind speed in an 11-day window around each day of the year. The resulting time series is then smoothed by a 30-day running mean. A climatology of the wind intensity is then calculated as the module of the zonal and meridional wind climatologies and removed from the time series of the module of the wind to obtain daily anomalies of the wind intensity on each point of the domain. Wind anomalies at the onset of a MHW are calculated by averaging wind anomalies on the first day of the event at any grid points located within 50 km of the MHW.

### El Niño index

The Coastal El Niño Index (ICEN) was established by the Commission in charge of the Study of the El Niño Phenomenon (ENFEN) for the diagnosis of El Niño and La Niña in Peru^46^. This index represents the variability of the regional climate in the eastern equatorial Pacific Ocean, which includes the areas off Ecuador and northern Peru. It is calculated as the three-month moving average of the SST anomaly in the Niño 1 + 2 region. More information is available at http://met.igp.gob.pe/variabclim/indices.html.

### Lagged correlations

To study the remote origins of MHWs, lagged correlations between MHW exposure at the equator and in the coastal strip are calculated. To this end we use the SST averaged in the equatorial box (between 100 and $105^{\circ}\,\hbox{W}$ and $2^{\circ}\,\hbox{S}$ and $2^{\circ}\,\hbox{N}$) and in the five coastal sub-domains ($2.5^{\circ }{-}6^{\circ}\,\hbox{S}$, $6^{\circ }{-}10^{\circ}\,\hbox{S}$, $10^{\circ }{-}14^{\circ}\,\hbox{S}$, $14^{\circ }{-}18^{\circ}\,\hbox{S}$ and $18^{\circ }{-}25^{\circ}\,\hbox{S}$). Lagged correlation are then calculated between the equatorial time series of MHWs occurrences and each coastal time series. Correlations are calculated using time series that only include MHWs of a certain duration range to compare equatorial influence on each category of events. Only the highest correlation and corresponding lag is indicated Fig.7a when it is significant (${p} < 0.05$).
